# Supporting Quality Integrated Care for Adolescent Depression in Primary Care: A Learning System Approach

**DOI:** 10.5334/ijic.7685

**Published:** 2024-02-01

**Authors:** Diana Sarakbi, Dianne Groll, Joan Tranmer, Rodger Kessler, Kim Sears

**Affiliations:** 1 Health Quality Programs, Queen’s University, Ontario, Canada; 2 Department of Psychiatry and Psychology, Queen’s University, Ontario, Canada; 3 School of Nursing and Department of Public Health Sciences, Queen’s University, Ontario, Canada; 4 Department of Family Medicine, University of Colorado, Colorado, United States; 5 School of Nursing and Health Quality Programs, Queen’s University, Ontario, Canada

**Keywords:** mental health, primary care, children and adolescents, depression, integrated care

## Abstract

**Background:**

Quality integrated care, which involves primary care and mental health clinicians working together, can help identify and treat adolescent depression early. We explored systemic barriers to quality integrated care at the provincial level in Ontario, Canada using a learning system approach.

**Methods:**

Two Ontario Health Teams (OHTs), regional networks designed to support integrated care, completed the Practice Integration Profile (PIP) and participated in focus groups.

**Results:**

The OHTs had a median PIP score of 69 out of 100. Among the PIP domains, the lowest median score was case identification (50), and the highest one was workspace (100). The focus groups generated 180 statements mapped to the PIP domains. Workflow had the highest number of coded statements (59, 32.8%).

**Discussion:**

While the primary care practices included mental health clinicians on-site, the findings highlighted systemic barriers with adhering to the integrated care pathway for adolescent depression. These include limited access to mental health expertise for assessment and diagnosis, long wait times for treatment, and shortages of clinicians trained in evidence-based behavioral therapies. These challenges contributed to the reliance on antidepressants as the first line of treatment due to their accessibility rather than evidence-based guidelines.

**Conclusion:**

Primary care practices, within regional networks such as OHTs, can form learning systems to continuously identify the strategies needed to support quality integrated care for adolescent depression based on real-world data.

## Introduction

Adolescence is a time of growth, learning, and identity development that poses higher risks for mental health conditions like major depressive disorder, also known as depression [1]. According to the World Health Organization (WHO), depression is one of the leading causes of illness and disability for adolescents [2]. In Canada, the prevalence rate for depression is at 11% for 15- to 24-year-olds [3,4]. Adolescent depression can interfere with academic performance and increase the risk of substance use and suicide [5,6]. The age of onset for depression usually begins after puberty when adolescents may be vulnerable to this mental health disorder due to a combination of biopsychosocial risk factors [7,8,9,10]. Therefore, adolescence is a critical time to screen for symptoms of depression [11].

Primary care providers play a key role in the early detection and management of adolescent depression [12,13,14]. The integrated care pathway for adolescent depression, from screening to follow-up care, includes psychotherapy and medication as treatment options with reassessments during the first three months to adjust the course of treatment as needed [15,16]. However, the literature highlights gaps in adhering to this pathway in primary care, including missed diagnosis, inadequate treatment, and lack of follow-up care [17,18,19]. Learning how to support quality mental health services in primary care could help improve the detection and treatment rates of adolescent depression [20,21,22,23].

In Canada, Ontario Health Teams (OHTs) are being formed in phases under the leadership of Ontario Health, a provincial government agency, to support a regional approach to integrated care across all sectors [24,25]. OHTs are defined as “groups of providers and organizations that are clinically and fiscally accountable for delivering a full and coordinated continuum of care to a defined geographic population” [25]. The first 24 OHTs were approved in December 2019, increasing to 57 OHTs as of October 2023. Delivery of integrated care for people with mental health disorders across the region, from primary care to specialized mental health services, is one of the priorities for OHTs [25].

In the primary care sector in Ontario, there is a shift to a team-based approach to care where primary care clinicians (e.g., family physicians and nurses) and mental health clinicians (e.g., psychiatrists, psychologists and social workers) work together to provide integrated care within the health care practice (e.g., family health teams, community health centres, nurse practitioner-led clinics) [26,27]. Providing quality mental health services in primary care, referred to as integrated care in this study, requires a common framework, from screening to follow-up care, in addition to funding mental health positions in primary care [28,29,30]. Having a structured approach for delivering quality integrated care may lead to better patient outcomes [31].

The Practice Integration Profile (PIP) is the first valid and reliable survey measuring the level of integration of mental health services in primary care using six domains derived from the Agency for Healthcare Research and Quality (AHRQ) Lexicon for Behavioral Health and Primary Care Integration [32]. They are: (1) routine screening to identify cases, (2) consistent workflow for assessing, diagnosing, and treating patients, (3) comprehensive clinical services including non-pharmacological treatment options, (4) collaborative workspace, (5) shared care between primary care and mental health clinicians supported by ongoing communication and shared decision-making, and (6) patient engagement and retention strategies [33,34]. Potential strategies were identified from the literature to support the PIP domains for adolescent depression such as staffing, funding and clinician training [35].

**Figure 1:**
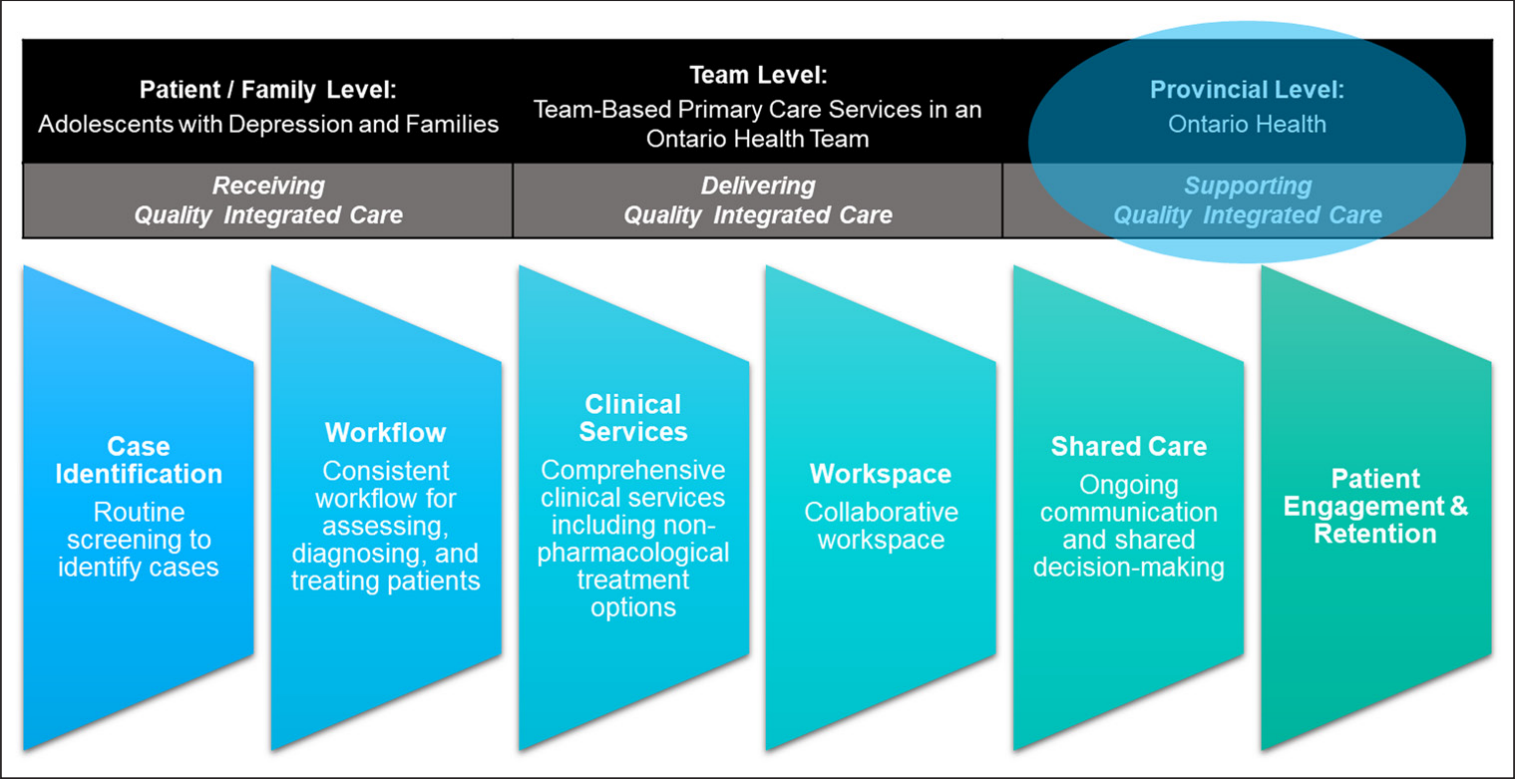
PIP Domains for Integrated Care at the Provincial Level [33,36].

Given the health reform led by Ontario Health, there was a policy window to research the support needed at the provincial level to provide quality integrated care for adolescent depression within a learning system framework (Figure 1). The aim of a learning system is to learn from every patient by leveraging real-world data generated at the point-of-care to continuously improve the quality of health services [36]. The purpose of this study was to explore how a learning system approach could support the ongoing recognition of systemic barriers and the strategies needed to support quality integrated for adolescent depression based on the real-world experiences of two OHTs.

## Methods

### Study Design

A mixed-methods design was used in this descriptive, multi-case study approved by the Queen’s University Health Sciences and Affiliated Hospitals Research Ethics Board. Four OHTs were identified as potential case sites in June 2022 because of their focus on child and youth mental health. Two of them agreed to participate in the study, referred to as OHT A and OHT B. Both OHTs had identified a community-based solution to support the early identification and treatment of mental health disorders in children and youth. Understanding the systemic barriers at the provincial level to quality integrated care focused on adolescent depression, a common mental health disorder in this population, would help support the quality improvement initiatives of these case sites.

### Data Collection & Analysis

#### Survey

The PIP (V1.0) consists of five demographic questions and 30 items grouped into six domains for integrated care [33,34]. The demographic questions were adapted to reflect the context in Ontario. Given the focus of this study was depression, references to “behavioral health” were replaced with “mental health”.

The PIP was distributed to all team-based primary care services in OHT A and OHT B. Three of the four primary care teams in OHT A completed the PIP for a response rate of 75%. The PIP was completed between November 21, 2022–January 18, 2023, by the nurse practitioner-led clinic, community health centre, and one of the two family health teams in OHT A. One of the two family health teams in OHT B completed the PIP between November 9, 2022–January 4, 2023, for a response rate of 50% at the OHT-level. This was a large, multi-practice family health team where 10 of its practice sites provided mental health services. Six of these 10 practice sites completed the survey for a response rate of 60% at the team-level. The family health team in OHT B that did not participate in the study had one dedicated mental health practice. Therefore, the total number of practices that completed the PIP from OHT A and OHT B was nine out of 15 eligible practices. This corresponded to a 60% response rate overall with 75% in OHT A (n = 3/4) and 55% in OHT B (n = 6/11).

A minimum of two people were invited to complete the survey on behalf of each primary care practice where feasible. The respondents were a mix of clinicians (nurse practitioners, family physicians, social workers, and psychiatrists) reflecting the team composition within these practices. The survey remained open for eight weeks. A reminder e-mail was sent one, three and seven weeks after the initial invitation e-mail [37]. A $50 gift card was awarded to one of the practices through a draw.

Descriptive statistics were used to summarize the demographic information of the primary care practices and the domain and overall scores (i.e., frequency, median and range, and mean and standard deviation). As per the PIP scoring rubric, each item received a score between 0–4 where 0 was “Never/None/0%” and 4 was “Always/All/100%”. The sum of these items was calculated for each domain (i.e., numerator), divided by the maximum score for that domain (i.e., denominator), and multiplied by a 100. Therefore, each domain received a score from 0 (no integration) to 100 (full integration). The total integration score was calculated using the mean of the six domain scores.

#### Focus Groups

A combination of purposeful and snowballing sampling was used to recruit focus group members from OHT A and OHT B in a representative role at the OHT-level where applicable. A total of 17 participants were recruited from OHT A and OHT B. The participants represented a variety of perspectives on quality integrated care, including administrators, primary care clinicians (family physician and nurse practitioners), mental health clinicians (social workers and psychiatrists), and a patient partner for youth mental health.

The principal investigator facilitated the focus group discussions. Each focus group was one hour and conducted virtually using Microsoft Teams ©. The focus group questions were open-ended and guided by the study’s conceptual framework. The first focus group was conducted on January 23, 2023 to test the clarity of the focus group guide with five participants from OHT A and OHT B. As the testing phase resulted in minimal revisions to the questions, these results were included in the analysis. Two focus groups were also conducted with six participants on February 2, 2023 (OHT A), and February 8, 2023 (OHT B). All the discussions were recorded and transcribed. Each participant was given a $25 gift card to thank them for their time.

The focus group transcripts were coded using NVivo 12 software (QSR International, Doncaster). A combination of deductive and inductive analysis was completed to find the overall “story” in the dataset [38,39]. First, the study’s conceptual framework was used to organize the focus group results, providing a codebook for coding the transcripts to the PIP domains and the learning system domain using content analysis. Second, a thematic analysis was completed to identify cross-cutting themes from all the transcripts using an iterative, inductive process in line with the study’s objectives.

Data triangulation was also used to analyze the results from multiple OHTs (i.e., OHT A and OHT B), perspectives (administrators, primary care clinicians, and mental health clinicians, and patient partner), and primary care teams (family health team, community health centre, and nurse practitioner-led clinic). A second reviewer validated the coded statements and independently coded a sample of the transcripts (10–25%), the equivalent of one domain selected at random. A summary of the findings was also shared with the participants for confirmation [40].

#### Methodological Triangulation

The survey and focus group results were analyzed together to form a more holistic perspective. The PIP median scores were compared to the number of coded statements by domain, as a high number of comments may reflect more concerns about a certain domain of integrated care. The PIP mean scores at the item-level were also compared to relevant quotes from the focus groups to determine whether they confirmed, complemented, or conflicted with the findings.

## Results

### Survey

#### Practice Demographics

OHT A and OHT B covered three types of primary care models (i.e., nurse practitioner led-clinics, community health centres, and family health teams) in various settings (i.e., inner city, urban, suburban, and rural areas). All nine primary care practices in OHT A and OHT B had been providing integrated care for more than a year and had at least one mental health clinician on the team, reflecting the characteristics of team-based primary care services in Ontario (Table 1).

**Table 1:** Practice Demographics (N = 9).


	CHARACTERISTIC	OHT A (n = 3)	OHT B (n = 6)

Practice Type	Community Health Centre	1	0

	Family Health Team	1	6

	Nurse Practitioner-Led Clinic	1	0

Practice Size	1–2 employees	0	0

	3–4 employees	0	0

	5–10 employes	0	0

	Greater than 10 employees	3	6

Practice Location	Inner City	0	2

	Urban	1	3

	Suburban	1	0

	Rural	1	1

	Frontier	0	0

Length of time integration effort has been active:	Effort is More Than 1 Year	3	6

The mental health clinician(s) in your practice is:	Employed by the practice or practice organization	3	6

How long has there been a mental health clinician as part of the practice?	More than 2 years	3	6


#### Domain and Overall Scores

**Figure 2:**
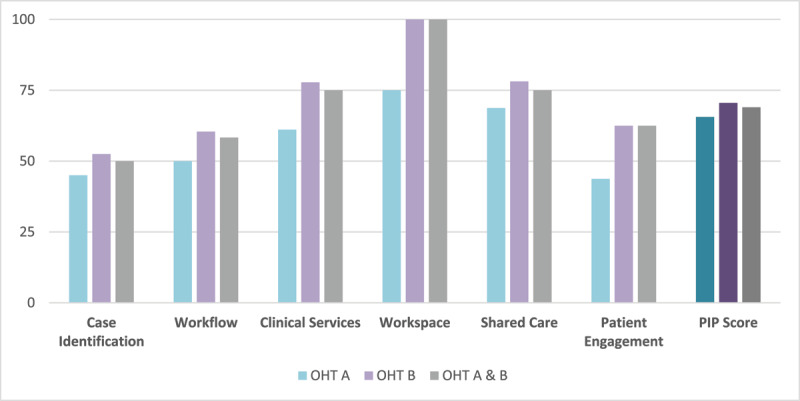
PIP Median Scores by Domain and Overall (N = 9).

The PIP scores for the primary care practices in OHT A and OHT B are presented at the OHT level in Supplementary Material 1 and at the item level in Supplementary Material 2. The nine primary care practices in OHT A and OHT B had a PIP median score of 69.0 out of 100.0 with a range of 46.4 to 80.0, and a PIP mean score of 68.8 out of 100.0 with a standard deviation of 10.0. The median domain scores ranked from lowest to highest were as follows: case identification (50.0), workflow (58.3), patient engagement and retention (62.5), clinical services (75.0), shared care (75.0), and workspace (100.0) (Figure 2). A similar ranking was obtained using the mean domain scores as follows: case identification (48.6), patient engagement and retention (58.9), workflow (60.9), shared care (73.8), clinical services (75.9), and workspace (94.4). The PIP domain with the highest median (100.0) and mean (94.4) scores for the nine practices was workspace. The PIP domain with the lowest median (50.0) and mean (48.6) scores was case identification.

### Focus Group

#### Themes

A total of 211 statements were coded from the transcripts. Most of these statements were coded to the PIP domains (180, 85.3%), while the rest were coded under learning system (31, 14.7%). Overall, these statements corresponded to the PIP domains except for workspace and shared care. None of the statements covered workspace, and the definition for shared care needed to be broadened from a collaboration between primary care and mental health clinicians within the same practice to partnerships between primary care, acute care, and community services from a system perspective, in line with the definition of OHTs.

**Figure 3:**
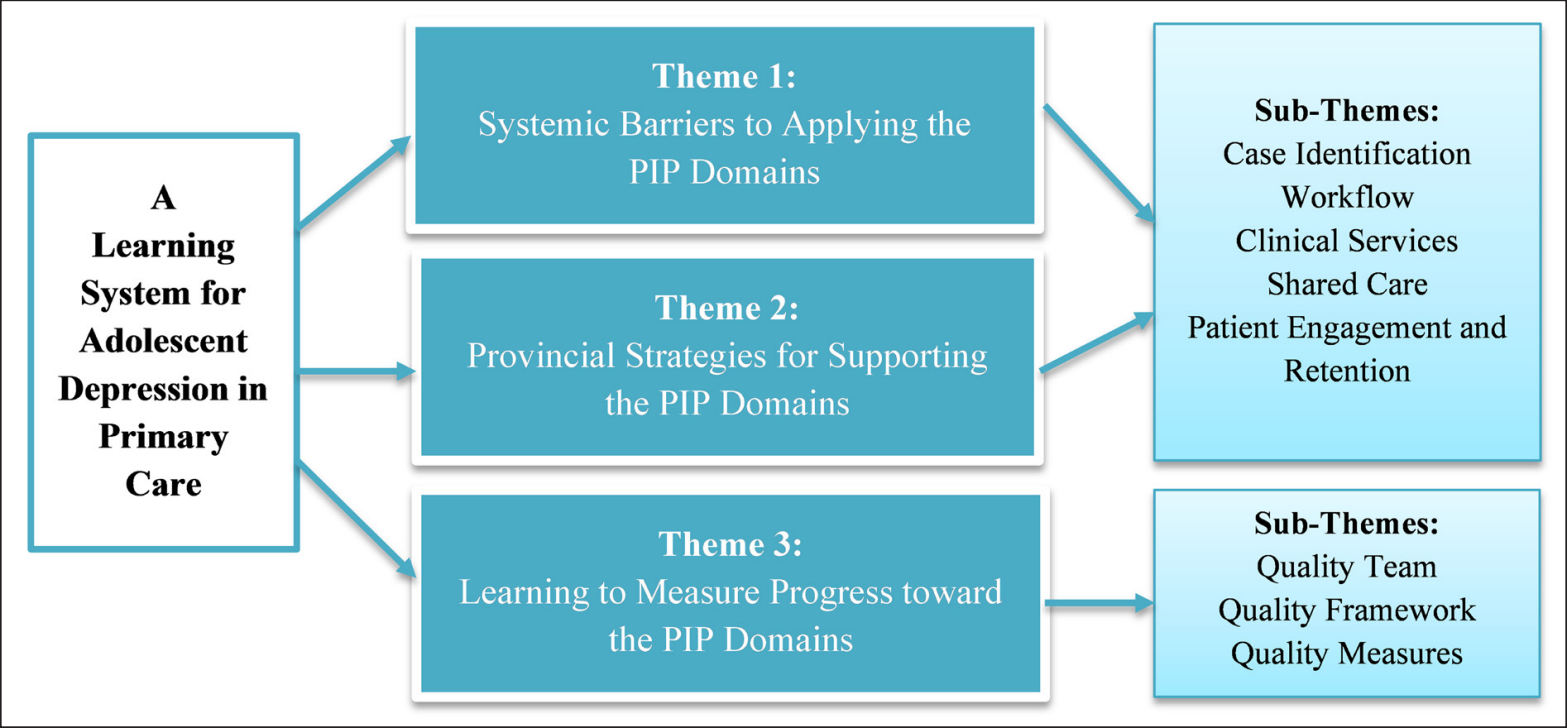
Conceptual Map of the Themes and Sub-Themes from the Focus Group Results.

As shown in Figure 3, the 211 statements were grouped under three themes to (1) identify the systemic barriers to quality integrated care for adolescent depression within each domain (98, 46.4%), (2) propose solutions for addressing these systemic barriers through supportive strategies within each domain (82, 38.9%), and (3) learn how to measure progress toward the domains (31, 14.7%). Excerpts from the transcripts are provided to illustrate the sub-themes with additional quotes in Supplementary Material 3.

#### Theme 1: Systemic Barriers to Applying the PIP Domains for Adolescent Depression

##### Case Identification

While participants shared their support for early identification of depressive symptoms, adolescents didn’t generally have annual well-visits for clinicians to complete the mental health screenings in primary care. There were also challenges with following-up on positive screens as one participant explained, “We have to be able to actually treat these patients once we identify them” (OHT B).

##### Workflow

Participants reported that the demand for mental health services exceeded the capacity of the teams to meet them, contributing to bottlenecks. Team capacity seemed to vary across practices as one participant highlighted, “We’re actually extremely lucky cause we’re very well-resourced with psychiatry compared to most family health teams” (OHT B). However, participants from OHT A and OHT B reported challenges with wait times for accessing mental health services within their teams and in the community. “The ones who don’t have benefits, they’ll have to rely on us if they don’t want to pay and so there’s going to be a waiting list” (OHT A).

Participants explained that depression is a complex mental health disorder where symptoms could be due to a variety of factors, including social issues. Primary care clinicians who didn’t feel comfortable diagnosing adolescents without consulting a mental health clinician referred them to external services for assessment, resulting in longer wait times. Therefore, some clinicians opted to complete a brief assessment to avoid delaying treatment. The prescription of medication was often the first line of treatment as explained by a participant, “By the time they’ve come to see me, the family doctors already tried one or two antidepressants without really being able to do a full diagnostic assessment. So, often the model already in play is purely biologic” (OHT B).

##### Clinical Services

Participants reported barriers to offering non-pharmacological treatment options for adolescent depression. As one participant indicated, “My approach was CBT first for mild-to-moderate adolescent depression, then antidepressants if failed CBT or if its severe and there’s suicidality, and because of the limitations in accessing psychotherapy, antidepressants are by and large first line” (OHT B).

One of the challenges included recruiting mental health clinicians trained in evidence-based therapies. “I know that’s a bigger picture thing, but I see that with the new grads, that they’re not trained in CBT, DBT, those types of therapies are effective” (OHT A). Another challenge was retaining mental health clinicians in primary care as they preferred to work in the acute care and private sectors for higher wages as highlighted by a participant, “You get them trained up and then they go take a higher paying job at the hospital and then you’re stuck training staff again” (OHT A). This challenge seemed to vary across practices in OHT B where one participant identified issues with recruiting “skilled mental health counselors” while another explained that “we’re able to implement those evidence-based strategies with our primary care colleagues.”

These challenges were perceived as contributing to a “two-tiered system” for mental health services where patient affordability rather than patient need informed the type of treatments available for adolescent depression. One participant reflected on this systemic barrier in the quote below.

“It is really frustrating in terms of the limitations in government funded psychotherapy access for children and teenagers, especially in the [OHT B] area where I don’t even refer to child and adolescent services anymore because the wait list is so long. It was a year before COVID and now I think it’s close to two years. So, I really essentially impress upon families that whatever they can afford in terms of private [services] and then also really work hard with the counselor within our office to try and have a few concentrated sessions. But you know, if an ideal CBT model is 12 to 16 weekly sessions and that’s what kids need to get that foundation, it just doesn’t exist” (OHT B).

##### Shared Care

The lack of investment in primary care was perceived as a foundational barrier to quality integrated care for mental health disorders. Participants reported that most of the resources were still being allocated to mental health services in the acute care sector. As a couple of participants stated, “I think to be fair to primary care it’s been inconsistently supported, and you’ve got hospital systems who have support for all of these kinds of things” (OHT B) and “Hospitals are trying to get youth out of the emergency department, but without the funding for the actual services in the community, it’s the hospitals continuing to get overloaded” (OHT A). Participants also identified the need for better coordination of care for patients from a system perspective between primary care, acute care, and community services.

##### Patient Engagement and Retention

Participants identified barriers for accessing primary care during school hours and transportation issues where parental support was needed, specifically in rural areas. While some depressed adolescents lacked the motivation to access mental health services, there didn’t seem to be a strategy for following up with adolescents who missed their appointments and/or weren’t adhering to their treatment plan. “I’ve had psychiatrists where if the patient isn’t willing to follow the medication recommendations that they’re getting, they discharge them. And like, there’s really no follow-up if they decide not to take the medication” (OHT A).

#### Theme 2: Provincial Strategies for Supporting the PIP domains for Adolescent Depression

##### Case Identification

Participants suggested screening adolescents at school using existing funded positions for mental health services, and in primary care when they come in for their immunization appointments between the ages of 14 and 16. Participants clarified that these patients needed to be screened for common mental health disorders and comorbidities, in addition to depression. This was reflected in the following response, “We don’t screen for depression on its own. The clinics that I work in, we use a child and youth screening tool” (OHT A). Participants also suggested considering sex and gender differences as part of the screening process with support from automated reminders.

##### Workflow

The first recommendation for improving workflow was increasing the number of mental health clinicians on the team to better meet demand. The second recommendation was to complete a comprehensive assessment of adolescents to understand what’s causing their symptoms of depression and recommend a course of treatment based on the results. This was highlighted in the response below from one of the participants.

“I mean someone has depression, but the cause of the depression can vary widely from abuse, neglect, to lack of housing, to lack of nutrition. So, depending on what causes the depression, it really could affect what treatment lines you go down” (OHT A).

This comprehensive assessment would expand treatment options instead of only relying on antidepressants because they’re more accessible as one participant explained, “I say comprehensive assessment only from the standpoint that you know you don’t want to medicalize something that’s being driven by something else in their life” (OHT B).

##### Clinical Services

Participants recommended funding training opportunities in evidence-based therapies. “Training the right people to do the right kind of thing is going to be critical because then it’s left to organizations to try and get people up to speed and that takes a long time. We need people now” (OHT B). Participants also suggested expanding treatment options based on evidence, including “social prescribing and bringing a more robust evidence base to some of that” (OHT B).

##### Shared Care

Participants recommended better coordination of care between primary care, acute care, and community services. As specified under workflow, a comprehensive assessment is needed for adolescent depression to better identify treatment options. This would require partnering with non-clinical community resources to include social services as reflected in the quote below.

“One of the things we’re trying to figure out with our mental health services review with the family health team is who needs straight up sort of mental health ongoing support for a mild-to-moderate condition versus psychiatric level care for kind of a moderate-to-severe problem versus more traditional social work” (OHT B).

A broader recommendation was to review the service delivery model of primary care to achieve better economies of scale by gathering more clinicians under a fewer number of practices and expand its team members to include patient navigators.

##### Patient Engagement and Retention

The first strategy was to help adolescents access mental health services by transporting them to the practice. The second strategy was offering virtual options to adolescents for treatment and follow-up visits, especially in rural areas. This option was perceived as less disruptive for adolescents because they wouldn’t “have to necessarily leave after-school job or school to have a doctor’s appointment” (OHT B). Participants also recommended partnering with high schools to provide on-site treatment to adolescents.

#### Theme 3: Learning to Measure Progress toward the PIP Domains

##### Dedicating Resources for Quality Improvement

Participants advised that a learning system for adolescent depression would need to be well-resourced for it to be implemented by the primary care team without adding to the workload of clinicians and take time away from patient care. As one participant explained, “We have to find that balance between making sure that we’re continuously learning and also making sure that it doesn’t become so burdensome for the clinicians that it becomes the focus of care, not the patient” (OHT B).

##### Having a Common Framework for Quality Integrated Care

Participants recommended having a common framework for quality integrated care and reviewing existing frameworks internationally that could be implemented in Ontario as explained by a participant, “I’m wondering if more work isn’t done to look at program systems around the world and what is working because we tend to reinvent the wheel which drives me insane” (OHT A). Participants suggested co-designing this framework with adolescents and holding education sessions with OHTs.

##### Leveraging Real-World Data

Participants supported the idea of learning from everyday data for quality improvement where one participant stated, “We need to create those living lab situations where we’re constantly learning, and it’s embedded as part of the protocols and practices” (OHT B). There was a recommendation to use a combination of data collection methods given the complexity of the primary care sector as explained in the following quote. “Primary care is a very multifaceted area of practice that requires a lot of really rich depth, qualitative, kind of thematic understanding about what it is that’s happening with the patient.” (OHT B). Participants also shared challenges with leveraging EHR to collect and share patient data with other primary care teams and community partners within the OHT given the variability of the information systems and the non-use of health cards in some of the community services.

### Integration of Results

Workflow had the highest number of coded statements (59/180, 32.8%), and the second lowest median score for OHT A and OHT B (58.3) as participants identified bottlenecks due to limited access to mental health expertise (Table 2). Whereas workspace had the highest median score for OHT A and OHT B (100.0) in line with the focus group findings where none of the statements were coded to this domain as mental health clinicians were funded to be part of the primary care team.

**Table 2:** PIP Scores and Number of Focus Group Statements by Domain.


PIP DOMAINS	MEDIAN SCORES	FOCUS GROUP STATEMENTS

Case Identification	50.0	37/180 (20.6%)

Workflow	58.3	59/180 (32.8%)

Clinical Services	75.0	21/180 (11.7%)

Shared Care*	75.0	19/180 (10.6%)

Workspace	100.0	0/180 (0%)

Patient Engagement and Retention	62.5	44/180 (24.4%)


*Revised definition from the PIP based on focus group results.

Table 3 includes examples of how the PIP scores confirmed, complemented, or conflicted with the focus group findings. The item with the lowest mean score under patient engagement and retention was having a strategy for following up with patients who didn’t adhere to their treatment plan (M = 1.3, SD = 1.1). This finding was supported by a participant who said, “There’s really no follow-up if they decide not to take the medication” (OHT A). While the item with the highest mean score under clinical services was having clinicians available on-site to provide mental health services (M = 3.8, SD = 0.5), participants explained that they didn’t have enough mental health clinicians on the team, resulting in long wait times. The item on prescribing medications for routine mental health conditions was one of the higher rated items (M = 3.6, SD = 0.5). However, participants highlighted that antidepressants were the first line of treatment for adolescents because they were more accessible than psychotherapy services.

**Table 3:** Comparing PIP Scores of OHT A and OHT B to Focus Group Quotes.


PIP ITEM	MEAN SCORE	FOCUS GROUP QUOTES

We have clinicians available on site who provide non-crisis focused mental health services (CS.1).	3.8	“So, I think that’s a whole barrier and sets an equity issue for kids that don’t have the opportunities as others for even treatment and care without psychiatry funded at an appropriate amount” (OHT A).

We offer prescription medications for routine mental health and substance abuse diagnoses (CS.7).	3.6	“You know, they have this limited insurance, so we really try and target treatment within what the parents can afford, because otherwise the options just don’t exist” (OHT B).

We have specific systems to identify and intervene on patients who did not initiate or maintain care (PE.3).	1.3	“I’ve had psychiatrists where if the patient isn’t willing to follow the medication recommendations that they’re getting, they discharge them. And like, there’s really no follow-up if they decide not to take the medication” (OHT A).


CS: Clinical Services/PE: Patient Engagement/OHT: Ontario Health Team.

## Discussion

### Interpretation of Results

The PIP scores of the primary care practices in OHT A and OHT B were compared to the scores obtained in the PIP validation study, recognizing that the comparison of these results is limited by the small sample size in the context of a case study (Table 4) [33]. The PIP was administered to primary care practices across the United States which included a group of practices without mental health clinicians (n = 20), practices with mental health clinicians (n = 102) and exemplars in integrated care (n = 8). The mean scores of the team-based practices in OHT A and OHT B (69) and in the validation study (60) were higher than the practices without mental health clinicians (27), but lower than the practices in the exemplar group (86). The ranking of the mean domain scores from lowest to highest was about the same between the two groups of team-based primary care practices, suggesting there could be similar challenges in applying the PIP domains which were further explored from a system lens in this case study.

**Table 4:** Comparing the PIP Mean Scores to the PIP Validation Study Results.


PIP QUALITY	CASE STUDY	PIP VALIDATION STUDY		
	
	TEAM BASED PRIMARY CARE (n = 9)	PRIMARY CARE (n = 20)	TEAM-BASED PRIMARY CARE (n = 102)	EXEMPLARS (n = 8)

Case Identification	49	43	53	83

Workflow	61	31	55	80

Clinical Services	76	23	67	94

Shared Care	74	18	56	86

Workspace	94	21	77	98

Patient Engagement and Retention	59	27	51	73

**Overall**	**69**	**27**	**60**	**86**


Overall, the focus group discussions complemented the PIP results by providing key insights that helped interpret the domain scores from a system perspective (Table 5). Limited access to mental health expertise seemed to be contributing to the reliance on antidepressants because they’re the most accessible form of treatment for adolescent depression. Strategies for addressing this systemic barrier included a comprehensive assessment of depressive symptoms, partnerships with high schools and social services to improve access to treatment options, and clinician training in evidence-based therapies for adolescent depression. A learning system framework that combines both quantitative (i.e., routine data from electronic health records) and qualitative (i.e., frontline experiences of clinicians and patients) data could help support the ongoing recognition of systemic barriers and the strategies needed to support quality integrated for adolescent depression.

**Table 5:** Key Learnings from the Multi-Case Study of OHTs.


	KEY LEARNINGS

Systemic Barriers	Limited access to mental health expertise within the primary care team and in the community is contributing to the reliance on antidepressants because they’re the most accessible form of treatment, specifically for adolescents without access to private insurance.

Strategies	Comprehensive assessments of depressive symptoms, partnerships with high schools and social services in the community and investing in training opportunities for evidence-based therapies could help improve treatment options for adolescent depression.

Learning System	A combination of quantitative and qualitative data is needed to support quality integrated for adolescent depression grounded in the frontline experiences of clinicians and patients.


### Application of Findings

Providing quality integrated care for adolescent depression goes beyond only funding mental health clinicians in primary care [28,29,30,31]. Integration efforts need to be guided by a common framework for quality at the national/sub-national level that is informed by real-world data. Therefore, we developed a learning system framework for quality integrated care focused on adolescent depression based on the study results. We included the perceived systemic barriers and proposed strategies by domain for integrated care. Suggested indicators were also included based on the PIP and the provincial standards for depression (Supplementary Material 4) [33,35,41]. Primary care practices, within regional networks such as OHTs, can build upon these results by forming a learning system to continuously identify the strategies needed for quality integrated care for adolescent depression based on real-world data.

#### Case Identification

Adolescents can be screened at school using existing funded positions for mental health services, and in primary care when they come in for their immunization appointments between the ages of 14 and 16 with support from automated reminders in the EHR platform [42]. A targeted indicator is tracking the number of adolescents screened for mental health disorders including depression in primary care.

#### Workflow

A comprehensive assessment can help understand what’s causing the adolescent’s symptoms of depression, identify goals of care and develop their treatment plan accordingly. This could be supported by clinical prompts in the EHR platform to assist clinicians in their decision-making process [43,44]. Depending on the assessment results, treatment options may include referral to non-clinical services in the community such as social services. This strategy could help alleviate some of the bottleneck issues in primary care by adopting a broader, system approach for social and mental health services with support from patient navigators [45].

A targeted indicator is tracking the number of adolescents who had received a comprehensive mental health assessment for their symptoms of depression in primary care. The timeframe for this assessment is based on the level of severity of the symptoms. For suspected severe depression within seven days of initial contact, and for suspected mild to moderate depression within four weeks of initial contact [41].

#### Clinical Services

The study results suggested that primary care treatment options for adolescent depression were limited. Non-pharmacological treatments were particularly challenging due to systemic barriers. The prescription of medication seems to be the first line of treatment for depression because it’s more accessible than psychotherapy services in Ontario. This is concerning because antidepressants seemed to be less effective for mild cases of adolescent depression and may increase risk of suicidality for this population [46,47,48].

Mild-to-moderate cases of mental health disorders could be treated in primary care with support from mental health clinicians so that specialized psychiatric services could be reserved for more severe cases [49]. For example, Cognitive Behavioral Therapy (CBT) could be provided for adolescent depression by either primary care or master-level clinicians with ongoing supervision by a mental health clinician [46,50,51,52,53]. It’s recommended to support training opportunities such as a certification course in CBT for adolescent depression [54]. The compensation models of mental health clinicians in primary care, such as social workers, may also need to be reviewed to ensure they are comparable to those of hospital-based mental health clinicians, as part of the provincial recruitment and retention strategy [55].

It’s recommended to track the number of adolescents who were offered evidence-based treatments for depression. For severe depression intervention should occur within seven days of the assessment, and for suspected mild to moderate depression within four weeks of the assessment. It is also recommend to monitor adolescents for an onset of, or increase in, suicidal ideation following the initiation of antidepressant medication [41].

#### Shared Care

This domain was revised to cover partnerships needed at the health system level to provide quality integrated care, including primary care, acute care, and community services. Primary care has a foundational role in building a public health system focused on preventive care by identifying and managing patients with mental health needs, however the study results suggested that most of the resources continue to be allocated to mental health services in the acute care sector [56,57]. In this perspective, it’s recommended increasing funding for mental health clinicians and training in primary care settings, forming partnerships with non-clinical resources in the community such as social services and high schools, and including a patient navigator [45]. Investing in patient registries for mental health services could help better assess the demand for child and youth mental health services and support resource allocation [58].

#### Patient Engagement and Retention

Transportation was identified as a challenge specifically in rural areas where adolescents relied on their parents to transport them to their appointments. It’s suggested to have strategies in place to facilitate access to mental health services by providing outreach services and virtual options for treatment and follow-up visits [59]. It’s also recommended to partner with high schools in the community to provide treatment to adolescents on-site [60]. A targeted indicator is tracking the number of adolescents who had completed their mental health treatment for depression. An outcome indicator is to track the percentage of adolescents with depression who show a decrease in their unmet needs over time [41].

### Limitations

As this was a case study, the results can not be generalized to other OHTs. A second limitation was that the PIP was designed to measure the level of integration of mental health services in primary care overall while this study focused on adolescent depression. However, the PIP provided a broader framework for identifying challenges specific to adolescent depression that could be addressed as part of the OHT’s overall strategy for quality integrated care.

### Future Directions

The scope of this research was purposely limited to team-based primary care services to allow for a more in-depth understanding of the strategies needed to support quality integrated care for adolescent depression in addition to having mental health clinicians on the team. The findings could help understand the type of support needed within existing team-based primary care services before scaling this model to other practices and/or researching models to support non-team based primary care services to provide quality integrated care.

## Conclusion

Investing in learning systems is increasingly recognized as necessary to support the implementation of strategic directions on quality grounded in real-world experiences. This study explored how a learning system could support the ongoing recognition of systemic barriers and the strategies needed to support quality integrated for adolescent depression. Primary care practices, within regional networks such as OHTs, can build upon these results by forming a learning system adapted to their context, regularly administer the PIP to track progress against the domains, and measure the impact of supportive strategies on the detection and treatment rates of adolescent depression over time.

## Additional File

The additional file for this article can be found as follows:

10.5334/ijic.7685.s1 Supplementary Materiales. Supplementary Materiales 1 to 4.

## References

[1] Uher R, et al. Major depressive disorder in DSM-5: implications for clinical practice and research of changes from DSM-IV. Depress Anxiety. 2014; 31(6): 459–71. DOI: 10.1002/da.22217 24272961

[2] World Health Organization. Health for the world’s adolescents. A second chance in the second decade. 2014.

[3] Statistics Canada. Table: 13-10-0465-01 Mental Health Indicators. 2014. DOI: 10.25318/1310046501-eng

[4] Findlay L. Depression and suicidal ideation among Canadians aged 15 to 24. Health Rep. 2017; 28(1): 3–11. 28098916

[5] Jaycox LH, et al. Impact of Teen Depression on Academic, Social, and Physical Functioning. Pediatrics. 2009; 124(4): e596. DOI: 10.1542/peds.2008-3348 19736259

[6] Thapar A, et al. Depression in adolescence. Lancet (London, England). 2012; 379(9820): 1056–1067. DOI: 10.1016/S0140-6736(11)60871-4 22305766 PMC3488279

[7] Wilson S, et al. Age of onset and course of major depressive disorder: associations with psychosocial functioning outcomes in adulthood. Psychological medicine. 2015; 45(3): 505–514. DOI: 10.1017/S0033291714001640 25007761 PMC4289461

[8] Lewinsohn PM, et al. Major depression in community adolescents: age at onset, episode duration, and time to recurrence. J Am Acad Child Adolesc Psychiatry. 1994; 33(6): 809–18. DOI: 10.1097/00004583-199407000-00006 7598758

[9] Maughan B, Collishaw S, Stringaris A. Depression in childhood and adolescence. Journal of the Canadian Academy of Child and Adolescent Psychiatry = Journal de l’Academie canadienne de psychiatrie de l’enfant et de l’adolescent. 2013; 22(1): 35–40. PMC3565713 23390431

[10] Hankin BL, et al. Development of depression from preadolescence to young adulthood: emerging gender differences in a 10-year longitudinal study. J Abnorm Psychol. 1998; 107(1): 128–40. DOI: 10.1037/0021-843X.107.1.128 9505045

[11] Canadian Paediatric Society. Age limits and adolescents. Paediatrics & child health. 2003; 8(9): 577–578. DOI: 10.1093/pch/8.9.577 20019831 PMC2794325

[12] Cheung A, Sinyor M. Depression in children and adolescents in primary care. Pediatric Medicine; Vol 4 (February 28, 2021); 2021. DOI: 10.21037/pm-20-82

[13] MHASEF Research Team. The Mental Health of Children and Youth in Ontario: 2017 Scorecard. Chart Pack. Toronto, ON: Institute for Clinical Evaluative Sciences; 2017.

[14] Kates N, et al. The Evolution of Collaborative Mental Health Care in Canada: A Shared Vision for the Future. Canadian Journal of Psychiatry. 2011; 56(5): I1–I10.

[15] Courtney D, et al. A Way through the woods: Development of an integrated care pathway for adolescents with depression. Early Intervention in Psychiatry. 2020; 14(4): 486–494. DOI: 10.1111/eip.12918 31883210

[16] Courtney DB, Bennett K, Szatmari P. The Forest and the Trees: Evidence-Based Medicine in the Age of Information. J Am Acad Child Adolesc Psychiatry. 2019; 58(1): 8–15. DOI: 10.1016/j.jaac.2018.06.035 30577942

[17] Olson AL, et al. Primary care pediatricians’ roles and perceived responsibilities in the identification and management of depression in children and adolescents. Ambul Pediatr. 2001; 1(2): 91–8. DOI: 10.1367/1539-4409(2001)001<0091:PCPRAP>2.0.CO;2 11888379

[18] O’Connor BC, et al. Usual Care for Adolescent Depression From Symptom Identification Through Treatment Initiation. JAMA Pediatr. 2016; 170(4): 373–80. DOI: 10.1001/jamapediatrics.2015.4158 26832387 PMC5541862

[19] Cloutier AM, et al. Effectiveness of risk communication interventions on the medical follow-up of youth treated with antidepressants. Psychiatry Res. 2013; 209(3): 471–8. DOI: 10.1016/j.psychres.2012.12.029 23664663

[20] Lewandowski RE, et al. Evidence for the management of adolescent depression. Pediatrics. 2013; 132(4): e996–e1009. DOI: 10.1542/peds.2013-0600 24043282 PMC4074649

[21] Asarnow JR, McKowen J, Jaycox LH. Improving care for depression: Integrating evidence-based depression treatment within primary care services. In: Treatments for adolescent depression: Theory and practice; 2009: 159–174. New York, NY, US: Oxford University Press. DOI: 10.1093/med:psych/9780199226504.003.0007

[22] Asarnow JR, et al. Integrated Medical-Behavioral Care Compared With Usual Primary Care for Child and Adolescent Behavioral Health: A Meta-analysis. JAMA Pediatrics. 2015; 169(10): 929–937. DOI: 10.1001/jamapediatrics.2015.1141 26259143

[23] Asarnow JR, Jaycox LH, Anderson M. Depression among youth in primary care models for delivering mental health services. Child Adolesc Psychiatr Clin N Am. 2002; 11(3): 477–97, viii. DOI: 10.1016/S1056-4993(02)00006-8 12222079

[24] Ontario Health. About Us; 2023.

[25] Ontario Ministry of Health and Long-Term Care. Ontario Health Teams: Guidance for Health Care Providers and Organizations; 2019.

[26] Sunderji N, et al. Evaluating the Implementation of Integrated Mental Health Care: A Systematic Review to Guide the Development of Quality Measures. Psychiatric Services. 2017; 68(9): 891–898. DOI: 10.1176/appi.ps.201600464 28502244

[27] Ontario Health. Ontario Health Teams – Primary Care Communications Protocol: Enabling Success through Connecting Primary Care and Physician Partners; 2021. Available from: https://health.gov.on.ca/en/pro/programs/connectedcare/oht/docs/OHT_primary_care_comms_protocols_guide.pdf.

[28] Goodrich DE, et al. Mental health collaborative care and its role in primary care settings. Current psychiatry reports. 2013; 15(8): 383–383. DOI: 10.1007/s11920-013-0383-2 23881714 PMC3759986

[29] Auxier A, et al. Behavioral health referrals and treatment initiation rates in integrated primary care: a Collaborative Care Research Network study. Transl Behav Med. 2012; 2(3): 337–44. DOI: 10.1007/s13142-012-0141-8 24073133 PMC3717910

[30] Unützer J, Park M. Strategies to improve the management of depression in primary care. Prim Care. 2012; 39(2): 415–31. DOI: 10.1016/j.pop.2012.03.010 22608874 PMC4127627

[31] Constance van E, et al. New Directions from Studying Integrated Behavioral Health and Primary Care for Patients with Multiple Chronic Conditions. The Annals of Family Medicine. 2023; 21(Supplement 1): 3807.

[32] Peek CJ. The National Integration Academy Council. Lexicon for Behavioral Health and Primary Care Integration: Concepts and Definitions Developed by Expert Consensus. 2013; 1–57.

[33] Kessler RS, et al. Development and validation of a measure of primary care behavioral health integration. Fam Syst Health. 2016; 34(4): 342–356. DOI: 10.1037/fsh0000227 27736110

[34] Mullin DJ, et al. Measuring the integration of primary care and behavioral health services. Health Serv Res. 2019; 54(2): 379–389. DOI: 10.1111/1475-6773.13117 30729511 PMC6407343

[35] Sarakbi D, et al. Achieving Quality Integrated Care for Adolescent Depression: A Scoping Review. Journal of Primary Care & Community Health. 2022; 13: 21501319221131684. DOI: 10.1177/21501319221131684 PMC9647275 36345229

[36] Sarakbi D, et al. Aiming for quality: a global compass for national learning systems. Health Research Policy and Systems. 2021; 19(1): 102. DOI: 10.1186/s12961-021-00746-6 34281534 PMC8287697

[37] Hoddinott SN, Bass MJ. The dillman total design survey method. Canadian family physician Medecin de famille canadien. 1986; 32: 2366–2368. 21267217 PMC2328022

[38] Potter WJ, Levine-Donnerstein D. Rethinking validity and reliability in content analysis. Journal of Applied Communication Research. 1999; 27(3): 258–284. DOI: 10.1080/00909889909365539

[39] Braun V, Clarke V. Using thematic analysis in psychology. Qualitative Research in Psychology. 2006; 3(2): 77–101. DOI: 10.1191/1478088706qp063oa

[40] O’Connor C, Joffe H. Intercoder Reliability in Qualitative Research: Debates and Practical Guidelines. International Journal of Qualitative Methods. 2020; 19: 1609406919899220. DOI: 10.1177/1609406919899220

[41] Ontario Health. Quality Standards – Major Depression Care for Adults and Adolescents; 2016.

[42] Sekhar DL, et al. Screening in High Schools to Identify, Evaluate, and Lower Depression Among Adolescents: A Randomized Clinical Trial. JAMA Netw Open. 2021; 4(11): e2131836. DOI: 10.1001/jamanetworkopen.2021.31836 34739064 PMC8571659

[43] Ellis LA, et al. Assessing the quality of care for paediatric depression and anxiety in Australia: A population-based sample survey. Australian & New Zealand Journal of Psychiatry. 2019; 53(10): 1013–1025. DOI: 10.1177/0004867419866512 31394909

[44] Hudson DL. Quality Over Quantity: Integrating Mental Health Assessment Tools into Primary Care Practice. Perm J. 2016; 20(3): 15–148. DOI: 10.7812/TPP/15-148 PMC4991920 27352418

[45] Bowles K, et al. Family and Youth Mental Health Needs and Outcomes in a Navigation Service: A Retrospective Chart Review. J Can Acad Child Adolesc Psychiatry. 2020; 29(4): 218–228. 33184566 PMC7595253

[46] Clarke G, et al. Cognitive behavioral therapy in primary care for youth declining antidepressants: A randomized trial. Pediatrics. 2016; 137(5): e20151851. DOI: 10.1542/peds.2015-1851 27244782 PMC4845864

[47] Hetrick SE, et al. Newer generation antidepressants for depressive disorders in children and adolescents. Cochrane Database Syst Rev. 2012; 11(11): Cd004851. DOI: 10.1002/14651858.CD004851.pub3 23152227 PMC8786271

[48] Zhou X, et al. Comparative efficacy and acceptability of antidepressants, psychotherapies, and their combination for acute treatment of children and adolescents with depressive disorder: a systematic review and network meta-analysis. The lancet. Psychiatry. 2020; 7(7): 581–601. DOI: 10.1016/S2215-0366(20)30137-1 32563306 PMC7303954

[49] Ontario Health Mental Health and Addictions Centre of Excellence. Mental Health and Addictions System Performance in Ontario 2021 Scorecard; 2021.

[50] Forman-Hoffman V, et al. Screening for Major Depressive Disorder in Children and Adolescents: A Systematic Review for the U.S. Preventive Services Task Force. Annals of Internal Medicine. 2016; 164(5): 342–349. DOI: 10.7326/M15-2259 26857836

[51] Mufson L, et al. Stepped care interpersonal psychotherapy treatment for depressed adolescents: A pilot study in pediatric clinics. Administration and Policy in Mental Health and Mental Health Services Research. 2018; 45(3): 417–431. DOI: 10.1007/s10488-017-0836-8 29124527 PMC5911397

[52] Shippee ND, et al. Effectiveness in Regular Practice of Collaborative Care for Depression Among Adolescents: A Retrospective Cohort Study. Psychiatric Services. 2018; 69(5): 536–541. DOI: 10.1176/appi.ps.201700298 29446330

[53] Richardson LP, et al. Collaborative care for adolescents with depression in primary care: a randomized clinical trial. JAMA: Journal of the American Medical Association. 2014; 312(8): 809–816. DOI: 10.1001/jama.2014.9259 25157724 PMC4492537

[54] Centre for Addiction and Mental Health. The CARIBOU Pathway by CAMH: An integrated care pathway for the treatment of youth depression; n.d.

[55] Ashcroft R, et al. The Emerging Role of Social Work in Primary Health Care: A Survey of Social Workers in Ontario Family Health Teams. Health & Social Work. 2018; 43(2): 109–117. 29490042 10.1093/hsw/hly003

[56] World Health Organization. Integrating mental health into primary care: a global perspective; 2008.

[57] Starfield B, Shi L, Macinko J. Contribution of primary care to health systems and health. Milbank Q. 2005; 83(3): 457–502. DOI: 10.1111/j.1468-0009.2005.00409.x 16202000 PMC2690145

[58] Ovretveit J, Nelson E, James B. Building a learning health system using clinical registers: a non-technical introduction. Journal of Health Organization & Management. 2016; 30(7): 1105–1118. DOI: 10.1108/JHOM-06-2016-0110 27700477

[59] Gilkey MB, et al. Using Telehealth to Deliver Primary Care to Adolescents During and After the COVID-19 Pandemic: National Survey Study of US Primary Care Professionals. J Med Internet Res. 2021; 23(9): e31240. DOI: 10.2196/31240 34406974 PMC8437399

[60] Zhang Q, Wang J, Neitzel A. School-based Mental Health Interventions Targeting Depression or Anxiety: A Meta-analysis of Rigorous Randomized Controlled Trials for School-aged Children and Adolescents. Journal of Youth and Adolescence. 2023; 52(1): 195–217. DOI: 10.1007/s10964-022-01684-4 36229755 PMC9560730

