# Supplementary material for: Supporting Quality Integrated Care for Adolescent Depression in Primary Care: A Learning System Approach
Source: Int J Integr Care. 2024 Feb 1;24(1):6. doi: 10.5334/ijic.7685 (PMC10836164; doi:10.5334/ijic.7685)
Supplement: Supplementary Materiales. — Supplementary Materiales 1 to 4. [file ijic-24-1-7685-s1.pdf]

1. PIP Scores by Domain and Overall at the OHT Level

Domain	OHT A (n=3)				OHT B (n=6)				OHT A and OHT B (N=9)			
	Median	Range	Mean	SD	Median	Range	Mean	SD	Median	Range	Mean	SD
**Case Identification**	45.0	32.5-70.0	49.2	19.1	52.5	20.0-65.0	48.3	17.8	50.0	20.0-70.0	48.6	17.0
**Workflow**	50.0	43.8-100.0	64.6	30.8	60.4	37.5-70.8	59.0	11.6	58.3	37.5-100.0	60.9	18.2
**Clinical Services**	61.1	58.3-97.2	72.2	21.7	77.8	63.9-97.2	77.8	11.5	75.0	58.3-97.2	75.9	14.4
**Shared Care**	68.8	39.1-87.5	65.1	24.4	78.1	68.8-93.8	78.1	9.5	75.0	39.1-93.9	73.8	15.7
**Workspace**	75.0	75.0-100.0	83.3	14.4	100.0	100.0-100.0	100.0	0.0	100.0	75.0-100.0	94.4	11.0
**Patient Engagement and Retention**	43.8	29.7-75.0	49.5	23.2	62.5	56.3-81.3	63.5	9.2	62.5	29.7-81.3	58.9	15.4
**Overall**	65.6	46.4-80.0	64.0	16.8	70.6	65.8-79.1	71.1	5.1	69.0	46.4-80.0	68.8	10.0

OHT: Ontario Health Team / SD: Standard Deviation

2. PIP Scores by Item for OHT A and OHT B

Domain	Item	OHT A (n=3)		OHT B (n=6)		OHT A and OHT B (N=9)	
		Mean	SD	Mean	SD	Mean	SD
Case Identification	We screen eligible patients for at least one mental health condition using a standardized procedure.	2.3	1.6	3.7	0.5	2.8	1.0
	We use practice-level data to screen for patients at risk for at least one complex or special need.	1.6	2.1	0.5	0.5	0.9	1.3
	Patients are screened at least annually for at least one mental health condition related to a chronic medical problem.	1.8	1.0	2.3	1.2	2.2	1.1
	Patients are screened at least annually for lifestyle or behavioral risk factors.	2.8	1.3	2.5	1.2	2.6	1.2
	Screening data are presented to clinicians prior to (or at) patient encounters with recommendations for patient care.	1.3	0.7	1.3	1.0	1.3	1.1
Workflow	We use a standard protocol to identify, assess, treat, and follow up patients who need or can benefit from integrated mental health.	2.8	1.1	3.0	0.0	2.9	0.6
	We use registry tracking to identify and follow patients with identified mental health issues.	2.4	1.9	0.2	0.4	0.9	1.5
	We coordinate clinical care and/or provide bidirectional communication for patients with mental health issues who would benefit from specialty services (not primary care).	2.3	1.6	3.0	1.1	2.8	1.3
	We connect patients with mental health issues to non clinical community resources.	2.9	1.0	2.7	1.0	2.8	1.0
	We provide referral assistance to connect patients to specialty mental health resources.	2.9	1.0	3.0	1.1	3.0	1.0
	We use a standard approach for documenting patients' self-management goals.	2.3	1.6	2.3	0.8	2.3	1.0
Clinical Services	We have clinicians available on site who provide non-crisis focused mental health services.	3.5	0.9	4.0	0.0	3.8	0.5
	We have clinicians available on site to see patients in mental crisis.	2.3	1.5	1.7	1.2	1.9	1.3
	We have mental health clinicians who can see seriously mentally ill and substance-dependent	3.4	1.0	3.7	0.5	3.6	0.7

Domain	Item	OHT A (n=3)		OHT B (n=6)		OHT A and OHT B (N=9)	
	patients.						
	We offer mental health interventions for patients with chronic/complex medical illnesses.	3.9	0.1	3.7	0.5	3.8	0.4
	We offer complex or specialized mental health therapies.	1.2	1.6	2.2	1.2	1.8	1.3
	We offer evidence-based substance abuse interventions.	1.7	2.1	2.2	1.5	2.0	1.6
	We offer prescription medications for routine mental health and substance abuse diagnoses.	3.8	0.3	3.5	0.5	3.6	0.5
	We offer prescription medications for serious complex co-occurring mental health and/or substance abuse diagnoses.	3.7	0.6	3.8	0.4	3.8	0.4
	We offer referral to non-clinical services outside of our practice.	2.5	1.5	3.3	0.5	3.1	1.0
**Shared Care**	Mental health and medical clinicians regularly and actively exchange information about patient care.	3.1	0.9	3.7	0.5	3.5	0.7
	There are regular educational activities including both mental health and medical clinicians.	2.4	1.4	2.5	1.0	2.5	1.1
	Mental health and medical clinicians regularly spend time together collaborating on patient care.	2.8	1.3	3.2	0.4	3.1	0.7
	Patients with mental health needs have shared care plans developed jointly by the patient, mental health and medical clinicians and updated over time.	2.1	0.9	3.2	0.4	2.8	0.8
**Workspace**	Mental health and medical clinicians work in…	3.0	1.0	4.0	0.0	3.7	0.7
	Patient treatment/care plans are documented in a medical record accessible to both mental health and medical clinicians.	3.7	0.6	4.0	0.0	3.9	0.3
**Patient Engagement and Retention**	We successfully engage identified patients in mental health services.	2.9	1.1	2.7	0.5	2.8	0.7
	We successfully retain patients in mental health services.	2.9	1.1	3.0	0.0	3.0	0.6
	We have specific systems to identify and intervene on patients who did not initiate or maintain care.	0.8	1.0	1.5	1.2	1.3	1.1
	We have follow-up plans for all patients whose mental health needs are resolved.	1.3	0.7	3.0	1.1	2.4	1.3

OHT: Ontario Health Team / SD: Standard Deviation

3. Additional Focus Group Quotes by Themes and Sub-Themes

Themes	Sub-Themes	Quotes
Systemic Barriers to Applying the PIP Quality Domains for Adolescent Depression	Case Identification	“I know we are going to miss a huge amount of people because we're not seeing them routinely” (OHT A).<br>“What are we as a system doing with that information? It's always helpful to do screening, but then what?” (OHT B).
	Workflow	“We have asked for more funding for mental health, more social workers, but of course we didn't get it” (OHT A).<br>“There are differing levels of depression. It could be a situational episodic or chronic situation where medication is warranted” (OHT B).<br>“It’ll probably be a long time before they see the psychiatrist, because with one day a week, it's not a full day” (OHT A).<br>“We're left to diagnose people and do all kinds of psych meds that should be done with psychiatry” (OHT A).
	Clinical Services	“I think part of what we're struggling with right now is with the explosion of private practice. We're having trouble recruiting seasoned, skilled mental health counselors” (OHT B).<br>“They pass them on to a therapist that doesn’t have really good training or doesn’t have much training, and our profession gets a bad rap from docs because they don't see the value sometimes” (OHT A).<br>“So, I think that's a whole barrier and sets an equity issue for kids that don't have the opportunities as others for even treatment and care without psychiatry funded at an appropriate amount” (OHT A).<br>“You know, they have this limited insurance, so we really try and target treatment within what the parents can afford, because otherwise the options just don't exist” (OHT B).
	Shared Care	There's no investment in primary care. So, youth mental health is a key gap” (OHT A).<br>“I'm thinking of the clients that come to our service who have been diagnosed with depression as an adolescent, who are then dropped by the primary care team because they've left it for whatever reason” (OHT B).
	Patient Engagement and Retention	“We're going to pull you out of school so that you can come and receive further treatment and support is a tough model, right?” (OHT B).<br>“The parents aren't able to get their kids to their appointments or the follow-up necessary” (OHT A).<br>“The nurse practitioner clinic in our two high schools is a very interesting model because it's part of the school system. It's a drop-in clinic, but the nurse practitioner works very closely with the two schools” (OHT B).

Themes	Sub-Themes	Quotes
Policies/Strategies for Applying the PIP Quality Domains for Adolescent Depression	Case Identification	“The schools have people in everyday and a lot of the symptoms that we're looking to screen for would manifest in academic difficulty and trouble at school” (OHT B).<br>“It strikes me that the 14-to-16-year immunization might be a point of contact that something like this could be done” (OHT A).
	Workflow	“I think salaried psychiatry is a must to help the vulnerable populations” (OHT A).<br>“We'd love to have a couple more social workers” (OHT A).
	Clinical Services	“I remember when I was doing my NP masters looking at some of the research around counseling services, all of those sorts of modalities for helping people with depression, and especially youths, and I'm finding that some of the group therapy was validated as excellent for this population. I know there are some youth options or youth group options available, but it's a rarity and it's hard to find” (OHT A).
	Shared Care	“So right now, the primary care model includes the most rudimentary clinical providers. But ultimately, that team needs to be a heck of a lot bigger. It needs to include that traditional social worker who's not here to provide you with sort of ongoing CBT, but rather do the kind of a global assessment and navigating work” (OHT B).
	Patient Engagement and Retention	“You'd have better luck at getting them back if you change it to a phone call appointment” (OHT A).
Learning to Measure Progress toward the PIP Quality Domains	Dedicating Resources for Quality Improvement	“People with operational evaluation kind of experience can do the job of running the actual service and evaluating it and helping it to adapt in real time, so that the doctors can show up and just be doctors” (OHT B).
	Having a Common Framework for Quality Integrated Care	“I think that’s the ideal situation if we truly want to learn is that we need to create those living lab situations where we're constantly learning, and it's embedded as part of the protocols and practices” (OHT B).
	Leveraging Real-World Data	“So, we're not collecting this information. We have other measures because the ministry has told us what measures were supposed to be collecting as family health teams, child and youth mental health is nowhere on their agenda for data collection from us” (OHT B).

4. Learning System for Quality Integrated Care focused on Adolescent Depression

CASE IDENTIFICATION

Definition: Early detection of mental health disorders, including symptoms of depression, using a validated assessment instrument.

Perceived Systemic Barriers:

- Inability to conduct routine mental health screenings of adolescents in primary care because they don't schedule annual well-visits.

Potential Strategies:

- Supporting high schools in the community to conduct routine mental health screenings to identify symptoms of depression early.
- Screening for common mental health disorders including depression during immunization visits (e.g., 14-16 years old).

Indicator(s):

- Numerator = # of adolescent screened for mental health disorders including depression / Denominator = # of adolescent seen in the practice.

WORKFLOW

Definition: Following evidence-based guidelines for assessment, diagnosis, and treatment of adolescent depression, including referral to non-clinical community resources and specialized mental health services if needed.

Perceived Systemic Barriers:

- Referral to external services for assessment with long wait times due to an insufficient number of mental health clinicians.
- Brief assessment of depressive symptoms and prescription of antidepressants due to the limited availability of mental health clinicians.

Potential Strategies:

- Supporting a comprehensive mental health assessment of adolescents to determine a course of treatment for depression through clinician training, access to tools and resources, and clinical prompts in the EHR platform.
- Providing guidance to clinicians on the non-clinical community resources available in the community and supporting adolescents to access these services using patient navigators.

Indicator(s):

- Numerator = # of adolescents receiving a comprehensive mental health assessment / Denominator = # of adolescents in need of mental health services for depression.

CLINICAL SERVICES

Definition: Having clinicians on-site to provide evidence-based treatments to adolescents with depression including non-pharmacological treatment options.

Perceived Systemic Barriers:

- Prescription of antidepressants as first line of treatment for adolescents because of the long wait times to see a mental health clinician.
- Ineffective treatment of depression due to lack of mental health clinicians trained in evidence-based therapies for depression.
- Challenges retaining mental health clinicians such as social workers in primary care after training them in evidence-based therapies because they move on to positions in the acute care and private sectors with higher wages.

Potential Strategies:

- Supporting training opportunities in evidence-based therapies for depression such as CBT and IPT.
- Reviewing compensation models in primary care for mental health clinicians.

Indicator(s):

- Numerator = # of adolescents offered evidence-based treatments for depression / Denominator = # of adolescents in need of mental health services for depression.

SHARED CARE

Definition: Having an integrated care model between primary care, acute care, and community services to provide person-centred care to adolescents with depression.

Perceived Systemic Barriers:

- Insufficient number of mental health clinicians on the team to meet the demand and complexity of cases presenting for mental health services.

Potential Strategies:

- Investing in patient registries to better assess the demand for mental health services with a focus on child and youth mental health, including referral patterns for adolescents with depression.
- Examining service delivery model for team-based primary care services to achieve better economies of scale, and form partnerships with non-clinical resources in the community such as social services and high schools.
- Reviewing funding models to incentivize collaboration and sharing resources between primary care and acute care services with a focus on health prevention and promotion.

Indicator(s):

- Numerator = # of adolescents with depression in mental health registries / Denominator = # of adolescents in need of mental health services for depression.

PATIENT ENGAGEMENT AND RETENTION

Definition: Having patient-centred strategies to initiate treatment and provide support through ongoing management and follow-up of adolescents with depression.

Perceived Systemic Barriers:

- Challenges attending appointments as adolescents rely on their parents for transportation, especially in rural areas, and would need to visit the primary care team during school hours.

Potential Strategies:

- Providing pick-up services for adolescents and offering virtual options for treatment and follow-up appointments.
- Partnering with high schools in the community to offer mental health services on-site.

Indicator(s):

- Numerator = # of adolescents completing a mental health intervention for depression / Denominator = # of adolescents who initiate a mental health intervention for depression.
- Numerator = # of adolescent patients with depression who show a decrease in their unmet needs over time / Denominator = # of adolescent patients who a initiate mental health intervention for depression.
